# Trends in Percutaneous Coronary Interventions in New South Wales, Australia

**DOI:** 10.3390/ijerph6010245

**Published:** 2009-01-12

**Authors:** Daminda P. Weerasinghe, Farhat Yusuf, Nicholas J. Parr

**Affiliations:** 1 Department of Cardiothoracic Surgery, Prince of Wales Hospital, Randwick, NSW, Australia; 2 Faculty of Business and Economics, Macquarie University, North Ryde, NSW, Australia; E-mails: f.yusuf@efs.mq.edu.au (F. Y.); nparr@efs.mq.edu.au (N. J. P.)

**Keywords:** Percutaneous coronary intervention, coronary artery disease, Australia

## Abstract

This is the first detailed study on percutaneous coronary intervention (PCI) in New South Wales (NSW), Australia. Hospital data for PCIs carried out between 1 July 1990 and 30 June 2002 are analysed. The study explores trends in PCI rates by selected socio-demographic factors, the utilisation of angioplasties *vis-a-vis* stents, emergency admissions, and selected coexisting conditions which determine the disease status of PCI patients. Logistic regression models are used to study the medical conditions that require both PCI and coronary artery bypass graft (CABG). The PCI rate has grown rapidly at 12.1% per annum, with a particularly rapid increase for persons aged 75+. The rate of multiple stent utilisation increased at 4.6% per annum. Pacific-born and Middle-Eastern-born patients are more than twice as likely as the Australian-born to have diabetes. Factors affecting failure of PCI requiring CABG include perforation and multi-vessel disease. PCI services in public hospitals need to be increased to facilitate the availability of these procedures to all segments of the population, as do targeted community-level programmes to educate high-risk groups in the control of heart diseases.

## 1. Introduction

The history of catheterisation goes back to 3000BC. Documented cardiac catheterisation began with Hales’s equine biventricular catheterisation in 1711. The development of cardiac catheterisation techniques could be attributed to: Frossmann in 1929; Cournand, Richard and others in 1950s; Sones in 1958; Dotter in 1963; and Judkins and Amplatz in 1967 [1, 2]. Further progress was made in 1977 by a German cardiologist A.R. Gruntzig, who invented balloon angioplasty [3]. The inventor of the coronary stent Charles Dotter together with Andrew Craig, invented an expandable stent made out of nitinol, the material that is commonly used in stents today [4]. It is estimated that almost two million percutaneous coronary interventions (PCI) have been performed worldwide, with an estimated increase of 8% annually [5].

Coronary angioplasty was first performed in Australia in 1980 [6], and since then the number of angioplasty procedures performed each year has increased rapidly. Based on 1998 data, Australia ranked 4^th^ among 18 OECD countries in terms of performing PCIs on patients with acute myocardial infarction (AMI) [7]. In Australia, a significant increase in the incidence of PCIs has coincided with a decline in the incidence of coronary artery bypass grafts (CABG) [8]. Studies have examined PCI procedure prevalence rates for Australia based on aggregate level data [8, 9]. The use of aggregate data reflects the non-availability of patient based data. These studies were unable to explore the structural determinants of PCI procedures and patient outcomes in order to develop community level targeted programmes and policies. There have been numerous studies to determine the structural determinants in ill health and methods of addressing inequalities [10–12]. Some studies have suggested that when diagnosis is used as the selection criteria, the individual may appear more than once in the data [13, 14]. This study attempts to use surgical procedure as the selection criteria to minimise the multiple admissions of the same patient for the same complication within a given year.

This is the first detailed study on PCIs in New South Wales (NSW), Australia. The study analyses the trends over a 12-years period in PCI by the age and gender of the patients undergoing this procedure. It explores trends in the utilisation of angioplasties *vis-a-vis* stents, in emergency admissions, and in selected coexisting conditions which determine the disease status of PCI patients. The variation in these co-existing conditions by country of birth (COB) is examined. Finally the likelihood of an episode of care involving the patient having to undergo both PCI and CABG procedures is analysed. The main research questions are: 1) whether the likelihood of a patient undergoing this combination of procedures has changed significantly over time; 2) whether there are associations with of age and gender; 3) whether there is a significant association with whether or not the patient had a stent; and 4) whether there are significant associations between whether a patient had both PCI and CABG in the same hospitalisation and cardiac tamponade, perforation, hypotension and multi-vessel disease.

The medical and health services issues arising from these analyses should include the identification of: 1) demographic groups (for example migrant communities) with particular need for education in the control of coronary artery disease (CAD); 2) how demographic trends (for example population aging) may contribute to future demands for PCIs of differing types; and 3) the predictors of rescue coronary bypass post PCI procedures. This study fills the vacuum in literature by assessing the structural determinants of PCI procedures in NSW to address criterion, as given above, which could be utilised to develop policies for the whole country.

## 2. Methods

The data on PCIs performed on NSW resident patients aged 30 years and over were extracted from the Department of Health’s Inpatient Statistics Collection (ISC) for the 12 financial years from 1990–91 to 2001–02 with appropriate ICD9-CM and ICD10-AM codes [15]. In Australia the financial year is from July 1 of calendar year to June 30 in the next calendar year. Relevant data on characteristics of the population were taken from the 1991, 1996 and 2001 population censuses. Estimated resident population data for the intercensal years were provided by the Australian Bureau of Statistics.

There could be some patients who had more than one PCI done within the same year. However, it was not possible to distinguish patients who experienced multiple episodes of hospitalisation, as there was no access to the unique identifiers such as medical record numbers, names and dates of birth which are needed for data matching. Nor were there indications of the first episode for a particular problem or readmission [16]. Thus the numerators for all rates presented in this study represent the number of “episodes of hospitalisation” rather than the “patients”. The admission status of patients was categorised as one of three types: emergency, planned (elective) and other.

The variations in coexisting conditions of PCI patients by COB (with Australian born as the control) were assessed using separate logistic regression models. Due to the complexity in interpretation of models, no multiple complications were assessed.

A logistic regression model was used to assess the associations between selected variables and the dependent dichotomous variable; whether patients had both PCI and CABG procedures in the same hospitalisation. The selected variables were the type of PCI, the year it was performed, age, gender, admission status, perforation, cardiac tamponade, hypotension and the existence of multi-vessel disease. The year was entered in the model as a continuous variable, and age in three groups: 30–59, 60–74 and 75+; all other variables were entered as dichotomous variables.

## 3. Results

A total of 55,831 patient episodes with PCIs were recorded in NSW over the 12-year period. Overall, the number of PCI procedures performed in NSW increased by 12.1% per annum, slightly lower than the 15.3% per annum increase for Australia as a whole [9]. The greatest increases in standardised rates for PCI rates were recorded between 1996–97 and 1997–98. This contrasts with the pattern for Australia of a rapid growth in the PCI utilisation rate between 1992–93 and 1994–95 and a considerable decrease in the growth rate in the following financial year [17].

**Figure 1.**
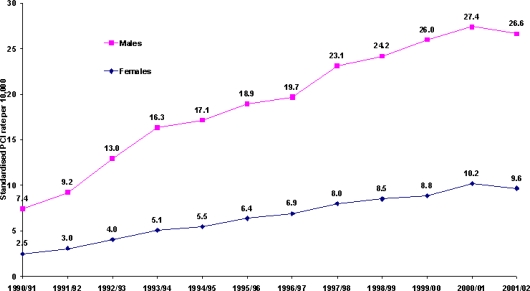
Age standardised PCI rates by gender, 1990–91 to 2001–02 (PCI rates for patients aged 30 years and above per 10,000, using the NSW resident population in December 1990 as the standard).

**Figure 2.**
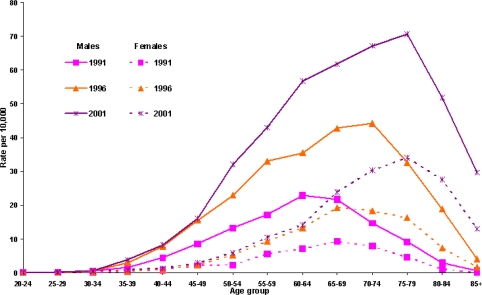
Age and gender specific PCI rates: NSW, census years 1991, 1996 and 2001 (PCI rates per 10,000 NSW residents age 30 years and above).

The PCI trends by gender in Figure 1 show the male rates were substantially higher those for females. While during most of the 1990s the gender gap seems to have widened, it seems to have narrowed somewhat in more recent years. An analysis of the age-specific rates for the three census years (1991, 1996 and 2001) shows that the modal age for PCI procedures for males increased from the 60–64 to the 70–74 age group over the 1991–96 period. However, there was no significant increase among women (Figure 2). Over the next five years, the modal age for both males and females increased to 75–79 years, however, the gender differential still persisted. While there was a steady decline in the age-specific PCI rates for males after the peak age, the decline was less steep for females.

**Figure 3.**
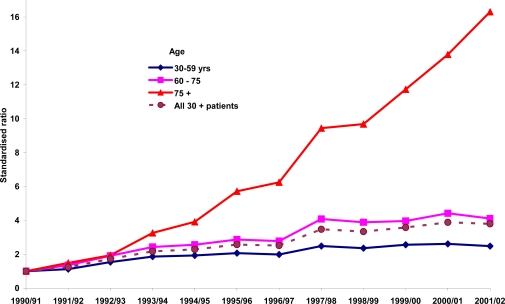
Indirectly standardised PCI ratios for selected age groups: NSW, 1990–91 to 2000–01 (1990–91 specific rates as the standard).

The PCI data were further analysed by subdividing into three broad age groups, 30–59, 60–74 and 75+ years, and calculating the standardised rates for each of the 12-years. Figure 3 indicates that while the gradient of increase for the youngest age group (30–59) was somewhat modest, it was steeper for the 60–74 age group, and was indeed sixteen-fold increase among people 75 years or older.

Over the 12-year study period, there was a steady increase in the median age of patients undergoing PCIs from 59.7 years in 1990–91 to 64.8 years in 2001–02. The ratios of males to females among the PCI patients were 5.1:1, 2.4:1 and 1.4:1 in the three age groups respectively. Two points are worth noting: firstly, that the gender gap in the performance of PCI procedures seems to have reduced significantly among the older people, and secondly, the large increase for older patients may be due in part to the advancement in skills of interventional cardiologists and improvements in technology as reported by Rentoukas *et al*. [18].

**Figure 4.**
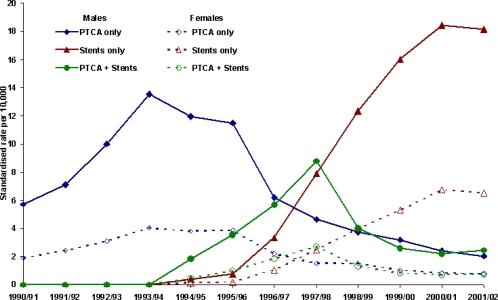
Age standardised PCI rates by type of procedure and sex, 1990–91 to 2001–02 (PCI rates per 10,000 NSW resident population aged 30+ in December 2001 as the standard).

Prior to 1994–95 stents were not coded separately in the NSW data and were probably included in the Percutaneous Transluminal Coronary Angioplasty (PTCA) data. Since then it has been possible to identify patients who had PTCA only, those who had stent(s) only and those who had both the PTCA and stent(s). It appears from Figure 4 that the use of PTCA only decreased substantially in recent years, while the use of stents increased dramatically. This was true for both males and females, but the female rates were consistently lower than the rates for males.

**Figure 5.**
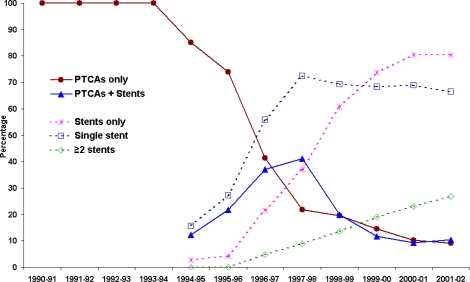
Trends in percentage of angioplasty and stent usage in NSW, 1990–91 to 2001–02 (Percentages are from total PCI episodes in each financial year).

Trends in the percentage of PCI patients who received PTCA and the number of stents over the study period are presented in Figure 5. These trends for NSW are similar to those for Australia as a whole [9]. Single and multiple stent data are available from the Inpatient database only from 1994–95. The proportion of patients receiving multiple stents increased at a rate of nearly 4.6% per annum. Since 1997–98 the decline in the percentage of single stents has been offset by an increase in the percentage of multiple stents. The highest stent utilisation was recorded in the last two study years 2000–02, over which time it remained steady at 91% of the total PCI episodes. This is similar to the stent utilisation pattern for all Australia [9].

**Figure 6.**
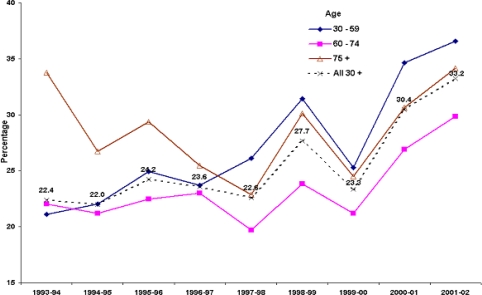
Percentage of PCI episodes which are emergency admissions by age group.

The admission status data were grouped into two categories: emergency and non-emergency, the latter including planned and other admissions. These data were available only from 1993–94 onwards (Figure 6). Over the nine-year period 73.3% of PCI admissions were non-emergency (68.6% planned and 4.7% ‘other’). Noticeable increases in the percentage of emergency patient episodes were recorded in the last two study years, with the increase being especially marked among younger patients (i.e. aged < 60). Over the rest of the study period the percentage of emergency admissions was relatively steady at around 24%. These results would reflect the difference in the preoperative status of PCI patients. Hence data were further assessed by the coexisting conditions of the PCI patients.

Data for coexisting conditions were extracted from the diagnosis variables (Table 1). It appears that hypertension, AMIs and diabetes were the most commonly reported coexisting conditions. The prevalence of most of these conditions appears to increase over time. This could partly be real and partly due to the changes in recording procedures over time. The Box-Jenkins auto regressions (with a 1 year lag) show that there was a statistically significant increase in the proportion of PCI cases who had diabetes, ARF, AMI and, to a lesser extent, hypertension.

The multivariate associations of coexisting conditions for six COB groups are given in Table 2. In comparison to the Australian-born, the prevalence of diabetes was highest among the Pacific-born PCI patients followed by the Middle-Eastern-born and the Asian-born. The Australian-born and the UK-born patients had a lower prevalence of diabetes. Compared to Australian-born patients, AMIs were significantly higher among the Pacific-born, Middle-Eastern-born and Asian-born PCI patients. Although the onset (pre or post procedure) of AMI cannot be determined based on the diagnoses data in the Inpatient database, for PCI patients it is most likely to be a pre-operative complication. UK-born patients had a significantly lower prevalence of AMI than the Australian-born. The prevalence of peripheral vascular diseases was highest among the UK-born and lowest among the Asian-born patients. Pulmonary disease was highest among the Australian-born and lowest among the Middle-Eastern-born patients. All migrant groups had lower pulmonary disease rates than the Australian-born. Compared to the Australian-born, significantly higher renal failure rates were recorded for Middle-Eastern-born and Asian-born patients. The variation in cerebrovascular disease was not significantly different between the Australian-born and the other COB groups.

In the data used for the current study, 885 (1.6%) patients were recorded as having undergone both PCI and CABG procedures in the same episode of care. A logistic regression model for these patients is presented in Table 3.

Although the gender effect was not statistically significant, female patients were slightly more likely than males to have both PCI and CABG procedures in the same episode of care. Studies have found that women may be at an increased risk of suffering PCI complications due to smaller vessel size [19, 20]. The age effect was significant at the 10% level. The coefficients show that, compared to the 60–74 age group, older patients were more likely to have both procedures, whilst younger patients were less likely to do so. The year of operation was entered in the model primarily to determine whether there was a change over time in the pattern of using both procedures in the same episode of care. Although there was no significant effect in the model, there was a slight tendency towards using more of both procedures over time. This could be as a result of improvements in PCI techniques that applied for patients with planned hybrid procedures involving both a PCI and a CABG.

Episodes with PTCA only were 7.4 times more likely to have both procedures than episodes with PTCA and stent combined or stent only. A high failure rate in angioplasties requiring CABG in the early years and a significant decline in the failure rate after the introduction of stents in Australia have been reported [9]. This is an indication of the high failure rate in PTCA only compared to PTCA + stent procedures, and not to the high utilisation of both PTCA only and CABG procedures. This effect also implies the low failure rate of stent only angioplasty requiring CABG procedures.

Although only a small proportion of patient episodes (0.68%) were identified with cardiac tamponade in the database, there was a significant effect on the dependent variable. Tseng *et al.* found that coronary artery perforation and delayed cardiac tamponade following balloon coronary angioplasty could occur several hours after discharge from the cardiac catheterisation laboratory [21]. Although there was a decline in cardiac tamponade post PCIs in NSW, studies suggest that the incidence of coronary perforation using new percutaneous revascularisation techniques may be increasing compared with PTCA [22, 23]. The authors recommend that patients should be observed for delayed cardiac tamponade for at least 24 hours.

Multi-vessel disease and perforation both have significant effects on the dependent variable. It is well established that the highest causes of failure of PCIs is the perforation of vessels followed by multi-vessel PCIs [21, 24, 25]. The logistic results show that the most significant factors leading to both procedures being performed in the same episode of care are the type of PCI and complications being occasioned by vessel perforation and attempted multi-vessel PCI. The effect of hypotension is not significant in the model.

## 5. Discussion

This paper shows the declining trend in the bypass rate [26] has been offset by the increasing trend in the PCI rate. There has been a steady increase in the median age of PCI patients from 59.7 to 64.8 years. The majority of younger patients were males, but among the older patients there was an increase in the proportion of female patients. These findings are consistent with previous studies [27, 28]. The introduction of stents has contributed to the dramatic increase in PCIs after 1995. Similar trends have been observed in Western Australia [29] and in the USA [30]. From a policy point of view, there is an increasing demand for PCI for women after menopause, hence, cardiac monitoring at regular intervals should be recommended for women especially after menopause.

In parallel with the increase in PCIs performed, there has been a significant increase (by almost 50%) in the percentage of emergency patients. Out of the selected co-existing complications of PCI patients, significant increases have been recorded for patients with AMI, diabetes mellitus and acute renal failure. An aging population, especially with the increasing percentage of patients with AMI, is likely to increase the percentage of emergency patients. These results are indicators of the applicability of PCIs for sicker patients who are unable to have these surgeries previously.

The increase in emergency patients could also be due to limitations in the current health care system, related to accessibility and the cost of health care [31]. The number of Medicare bulk-billed general practitioner and specialist care services are declining [32], so most consumers incur out of pocket costs above the scheduled fee for visits to private specialists [31]. Despite the Australian Government’s Pharmaceutical Benefits Scheme (PBS), which provides affordable access, medical costs can still present a barrier for some patients, particularly the chronically ill and those on lower incomes who are not eligible for government concessions [33]. Therefore due to poor management of CAD and limitations in the accessibility of hospital services, only critically ill patients may have been admitted to public hospitals. Further studies are needed to identify more precisely the reasons for limitations in resources and service delivery in the NSW health system.

Of the co-existing conditions, a significantly higher prevalence of AMI was recorded among the Pacific born patients. In addition, compared to Australian born, Pacific born patients are more likely to have diabetes. In the years to come, Pacific and Middle Eastern born people are likely to have an increasing risk of CAD requiring surgical intervention. In order to control the progression of CAD among ethnic communities in NSW, targeted community level education and prevention programs could be conducted. The PCI rates in NSW were found to be only marginally lower than the national average, our analysis has shown that the PCI procedures are becoming increasingly popular in an aging population with multiple co-morbidities. The PCI services in public hospitals need to be increased to further facilitate the availability of these procedures to all segments of the population, and to target community level programs to educate high-risk groups, in the control of heart diseases.

Over the period from 1990 to 1999 in Australia, on average 1.6% of angioplasty failure or complications required CABGs [9]. The NSW figure could contain all three possibilities for having both procedures: the hybrid PCI and CABG, CABG for PCI failure, and PCI after CABG. A US study reported that 27% of cases are for planned hybrid procedures involving both procedures [34]. The same study reported that the incidence of rescue PCIs following failed CABG has increased from 0% in 1994 to 1.6% in 2000. If the same proportion found in US data is applied to the NSW data from 1990 to 2001, the utilisation of rescue PCI following CABG should be for about 14 cases, which would be considered a relatively rare occurrence.

The patients with pre-planned hybrid procedures can be identified from a medical records review. Further studies could be done to determine the factors related to the decision of the hybrid procedures. Since hybrid procedures were introduced in the late 1990s [35] and this study is based on data applying to the period mid 1990 to mid 2002, the majority of the episodes recorded with both procedures would be for CABG after a PCI, rather than the hybrid procedure or the PCI post CABG.

Cardiac tamponade is a rare life-threatening situation, which was found to be highly significant in the logistic model in terms of whether patients had both procedures in the same episode of care. It is more likely to be indicative of a post PCI rescue CABG than the other way round. The logistic model showed the highest failures of PCIs were to the perforation of vessels followed by multi-vessel PCIs, a result supported by the findings of other studies [24, 25]. Therefore the focus should be on reducing the incidence of perforation of vessels and complications in multi-vessel PCIs.

### Competing interests

The authors declare that they have no competing interests

### Authors’ contributions

DPW designed the study, extracted data from the New South Wales Department of Health compiled large Inpatient Statistics Collection database, performed statistical analyses, literature search and written the manuscript. FY and NJP edited the manuscript. All authors read and approved the final manuscript.

## Figures and Tables

**Table 1.** Coexisting conditions in PCI patients: NSW, 1990–91 to 2000–01.

Financial year	Percentages of coexisting conditions^a^								
	Diabetes	ARF	CRF	AMI	CHF	Hypertension	PVD	CVD	PD
1990–91	8.2	0.1	1.0	8.0	2.0	25.1	2.8	1.5	3.6
1991–92	9.3	0.2	0.9	12.5	3.1	30.8	2.5	1.7	5.3
1992–93	8.4	0.1	0.7	12.6	3.7	28.6	3.0	1.1	4.6
1993–94	9.6	0.3	0.7	11.6	2.7	29.0	4.0	1.4	5.0
1994–95	10.0	0.2	0.5	13.9	2.8	28.8	3.0	1.3	4.8
1995–96	11.8	0.4	0.8	14.5	2.2	31.1	3.6	1.5	5.8
1996–97	12.3	0.3	1.1	19.4	2.1	35.7	3.6	1.2	7.0
1997–98	12.2	0.4	1.3	17.7	2.2	31.4	3.8	1.1	6.5
1998–99	13.6	0.4	1.4	20.1	2.8	35.0	3.5	1.3	7.0
1999–00	14.1	0.5	1.8	22.1	3.2	35.4	3.5	1.3	5.7
2000–01	16.4	0.6	1.7	26.2	3.5	39.7	3.1	1.0	4.8
2001–02	16.8	0.8	2.6	29.1	3.5	43.0	4.3	1.5	4.3
Overall	13.4	0.4	1.4	20.2	3.0	35.5	3.6	1.3	5.7
t value	11.4	7.0	3.1	8.6	1.0	5.3	1.5	–0.4	0.5
*p* value	<0.001	<0.001	0.011	<0.001	0.335	0.004	0.177	0.701	0.654

a Percentages of coexisting were derived from the total PCI patients in each financial year. The t and *p* values for the trend in specific coexisting conditions are from lag 1-year Box-Jenkins linear auto regression models. The independent and the dependent variables were financial year and the individual characteristic, respectively. Disease categories were: ARF, acute renal failure; CRF, chronic renal failure; AMI, acute myocardial infarction; CHF, Congestive heart failure; CVD, cerebrovascular disease; PD, pulmonary disease.

**Table 2.** Multivariate association of co-existing conditions of PCI patients by country of birth.

Independent variable	Logistic regression models by complications													
	Diabetes		RF		AMI		CHF		PVD		CVD		PD	
	OR	CI	OR	CI	OR	CI	OR	CI	OR	CI	OR	CI	OR	CI
Asia	***1.90 1.67–2.15***		***1.64 1.19 2.27***		***1.32 1.18 1.49***		1.23 0.91 1.65		***0.38 0.24 0.59***		0.88 0.54 1.43		***0.68 0.53 0.87***	
Europe	***1.59 1.48–1.70***		1.07 0.89 1.29		1.00 0.94 1.07		***1.45 1.27 1.65***		1.03 0.90 1.18		0.89 0.71 1.11		***0.71 0.63 0.81***	
UK	***0.72 0.65–0.81***		0.78 0.59 1.02		***0.83 0.76 0.91***		0.81 0.65 1.00		***1.21 1.03 1.41***		0.73 0.53 1.00		0.85 0.73 0.98	
Middle East	***2.45 2.18–2.76***		***1.67 1.20 2.31***		***1.34 1.19 1.50***		***1.58 1.21 2.06***		0.91 0.68 1.23		1.25 0.82 1.90		***0.66 0.51 0.85***	
Pacific	***3.02 2.43–3.75***		1.75 0.92 3.30		***1.37 1.10 1.71***		1.29 0.72 2.30		0.99 0.56 1.77		1.36 0.60 3.06		0.61 0.36 1.04	
Other	1.19 1.02–1.39		1.20 0.80 1.78		***1.35 1.19 1.52***		0.87 0.60 1.26		0.77 0.54 1.08		0.98 0.59 1.62		0.78 0.60 1.01	
Australia														
Sex - Male Female	***0.77 0.73–0.81***		0.96 0.83 1.10		0.99 0.95 1.05		0.73 0.66 0.81		***0.86 0.78 0.95***		***0.77 0.66 0.90***		***0.84 0.77 0.91***	
Age 30–59 60–74	***0.83 0.78–0.87***		***0.43 0.36 0.51***		***1.54 1.47 1.61***		***0.51 0.45 0.58***		***0.40 0.36 0.45***		***0.33 0.27 0.41***		***0.64 0.58 0.69***	
>=75	***0.90 0.83–0.97***		***2.71 2.35 3.12***		***1.26 1.18 1.35***		***2.27 2.02 2.55***		***1.63 1.46 1.82***		***1.48 1.24 1.77***		***1.27 1.15 1.40***	
c statistic	0.585		0.675		0.564		0.661		0.645		0.659		0.591	

RF, renal failure; AMI, acute myocardial infarction; CHF, congestive heart failure; PVD, peripheral vascular disease; CVD, cerebrovascular disease; PD, pulmonary disease, OR: Odds ratio, CI: 95% confidence interval, Numbers in bold italic denote significance at 0.05 level.

**Table 3.** Bivariate and multivariate association of the selected variables with the dependent variable being PCI and CABG procedures in the same episode of care.

Independent variable		% *	Bivariate		Multivariate	
			χ^2^	*p* value	OR (95% CI)	*p* value
Sex (female)		29.0	4.8	0.027	1.098 (0.943–1.279)	0.226
Age	30–59	37.7	2.9	0.083	0.875 (0.755–1.014)	0.076
	60–74	49.5	3.7	0.052		
	>=75	12.8	0.12	0.724	1.037 (0.836–1.287)	0.741
Year of PCIs (continuous variable)					1.011 (0.984–1.040)	0.424
Emergency admissions		52.9	112.5	<0.001	1.745 (1.509–2.018)	<0.001
PTCA only		73.6	381.9	<0.001	7.476 (5.947–9.398)	<0.001
PTCA & Stent combined and Stent only		26.4	121.7	<0.001		
Cardiac tamponade		0.68	20.2	<0.001	4.298 (1.719–10.74)	0.001
Perforation		7.8	418.9	<0.001	11.261 (8.506–14.91)	<0.001
Hypotension		3.5	60.9	<0.001	0.778 (0.105–5.786)	0.806
Multi-vessel disease		16.8	26.2	0.067	5.522 (4.340–7.027)	<0.001

* Percentages are from 885 episodes recorded with both PCI and CABG, OR=odds ratio, CI = confidence interval, c statistic = 0.736
